# *RARβ2* hypermethylation is associated with poor recurrence-free survival in never-smokers with adenocarcinoma of the lung

**DOI:** 10.1186/s13148-015-0066-4

**Published:** 2015-03-19

**Authors:** Yujin Kim, DongHao Jin, Bo Bin Lee, Eun Yoon Cho, Joungho Han, Young Mog Shim, Duk-Hwan Kim

**Affiliations:** Department of Molecular Cell Biology, Samsung Biomedical Research Institute, Sungkyunkwan University School of Medicine, #300 Chunchun-dong, Jangan-Ku, Kyunggido, Suwon 440-746 South Korea; Department of Pathology, Samsung Medical Center, Sungkyunkwan University School of Medicine, #50 Ilwon-dong, Kangnam-Ku, Seoul 135-710 South Korea; Department of Thoracic and Cardiovascular Surgery, Samsung Medical Center, Sungkyunkwan University School of Medicine, #50 Ilwon-dong, Kangnam-Ku, Seoul 135-710 South Korea; Samsung Biomedical Research Institute, Rm B155, #50 Ilwon-dong, Kangnam-Ku, Seoul 135-710 South Korea

**Keywords:** Lung cancer, RARβ2, Methylation, Never-smoker, Recurrence

## Abstract

**Background:**

This study was aimed at investigating if the effect of *RARβ2* hypermethylation on recurrence-free survival (RFS) in non-small cell lung cancer (NSCLC) depends on one’s smoking status and specific interacting proteins.

**Results:**

We retrospectively analyzed the expressions of five proteins using immunohistochemistry in archival formalin-fixed and paraffin-embedded tissues from 578 NSCLC patients who had undergone surgical resection from 1994 through 2004. Promoter methylation of *RARβ2* was assessed by bisulfite pyrosequencing. Recurrence was found in 268 (46%) of 578 NSCLCs with a median follow-up period of 4.8 years. Overexpression of β-catenin, c-MET, cyclin D1, and EGFR occurred in 55%, 72%, 51%, and 41% of the patients, respectively. E-cadherin expression was negative in 62% of the patients, and *RARβ2* hypermethylation was found in 37%. The abnormal expression of *c-MET* (*P* = 0.002) and *EGFR* (*P* = 0.001) was found to be highly prevalent in never-smokers. *RARβ2* hypermethylation was significantly associated with poor recurrence-free survival (RFS) in 128 never-smokers with adenocarcinoma (*P* = 0.01) For parsimonious model building, the five proteins were clustered into three groups (β-catenin and E-cadherin; c-MET; cyclin D1 and EGFR) by an unsupervised hierarchical clustering and were included in a multivariate analysis. Cox proportional hazard analysis showed that *RARβ2* hypermethylation was significantly associated with poor RFS in 128 never-smokers with adenocarcinoma (adjusted hazard ratio [HR] = 2.19, 95% confidence interval [CI] = 1.28 to 3.47; *P* = 0.009), after adjusting for interacting proteins.

**Conclusions:**

The present study suggests that *RARβ2* hypermethylation may be an independent prognostic factor of RFS in never-smokers with adenocarcinoma of the lung.

## Background

Despite significant advances in diagnosis and treatment over the past two decades, lung cancer is the leading cause of cancer-related death in the world, and the overall five-year survival rate remains under 15% [1,2]. The poor prognosis of lung cancer is partially due to the high rate of recurrence after surgical resection. Given that some patients may benefit from postoperative adjuvant therapy shortly after resection, the discovery of biomarkers for the identification of patients who are at a high risk of recurrence has become increasingly important for novel targeted therapy and for improving recurrence prevention. In recent years, a number of genetic and epigenetic molecules have been discovered for the purpose, and novel therapeutic agents are being developed that target those molecules.

Retinoic acid (RA) induces growth inhibition and apoptosis mainly by regulating gene expression through its nuclear receptors, known as retinoic acid receptors (RARs) and retinoid X receptors (RXRs). The receptors bind to a retinoic acid response element (RARE), consisting of two 5′-AGGTCA-3′ direct repeats of the consensus half-site sequence 5′-AGGTCA-3′, in the promoter region of the target genes to initiate their transcription. Both RAR and RXR receptors have three subtypes (α, β, and γ) which have distinct functions. Of these receptors, the retinoic acid receptor beta (RARβ) is expressed primarily in epithelial cells and plays a central role in mediating the growth inhibition of different types of cancer cells by retinoic acid. The loss and reduction of RARβ expression have been reported in a large percentage of patients with lung cancer. The human RARβ gene has four isoforms (β1, β2, β3, and β4), and the β2 isoform is most abundant among the isoforms. In addition, several studies have indicated that RARβ2 is primarily responsible for limiting the growth of lung cancer cells [3-5]. Indeed, expression of RARβ2 in RARβ-negative lung cancer cells has been shown to restore retinoic acid-induced growth inhibition [3], and transgenic mice expressing antisense or other constructs that down-regulate RARβ2 developed lung cancer [4].

The aberrant methylation of CpG islands at a promoter region of tumor suppressor genes is an epigenetic change that induces the transcriptional silencing of a gene. The *RARβ2* promoter contains a CpG island in the 5′-untranslated region that is frequently methylated in cancer cells. Abnormal hypermethylation of the *RARβ2* CpG island has been found in approximately 40% of non-small cell lung cancers (NSCLCs) and identified as part of a mechanism by which RARβ2 expression is repressed [6-8]. In contrast to many previous reports about the tumor-suppressive effect of RARβ in lung cancer, Kurie et al. [9] reported a negative effect of RARβ expression on overall survival of stage I NSCLC. We also reported a protective effect of *RARβ2* hypermethylation on the development of second primary lung cancers (SPLCs) in smokers with NSCLC [8]. Based on these reports, we hypothesized that an effect of *RARβ2* hypermethylation on the recurrence in NSCLC may differ according to exposure to tobacco smoke.

Cyclin D1, β-catenin, and E-cadherin are known to interact with RAR [10-18], and the altered expression of EGFR is known to occur at a high prevalence in never-smokers [19,20]. In an effort to investigate whether *RARβ2* hypermethylation contributes to the development of recurrence in NSCLC according to statuses of smoking and interacting proteins, we analyzed the methylation status of *RARβ2* and the expression statuses of cyclin D1, E-cadherin, β-catenin, EGFR, and c-MET in 578 NSCLC patients.

## Results

### Correlation of expression levels among five proteins differs according to smoking status

**Figure 1:**
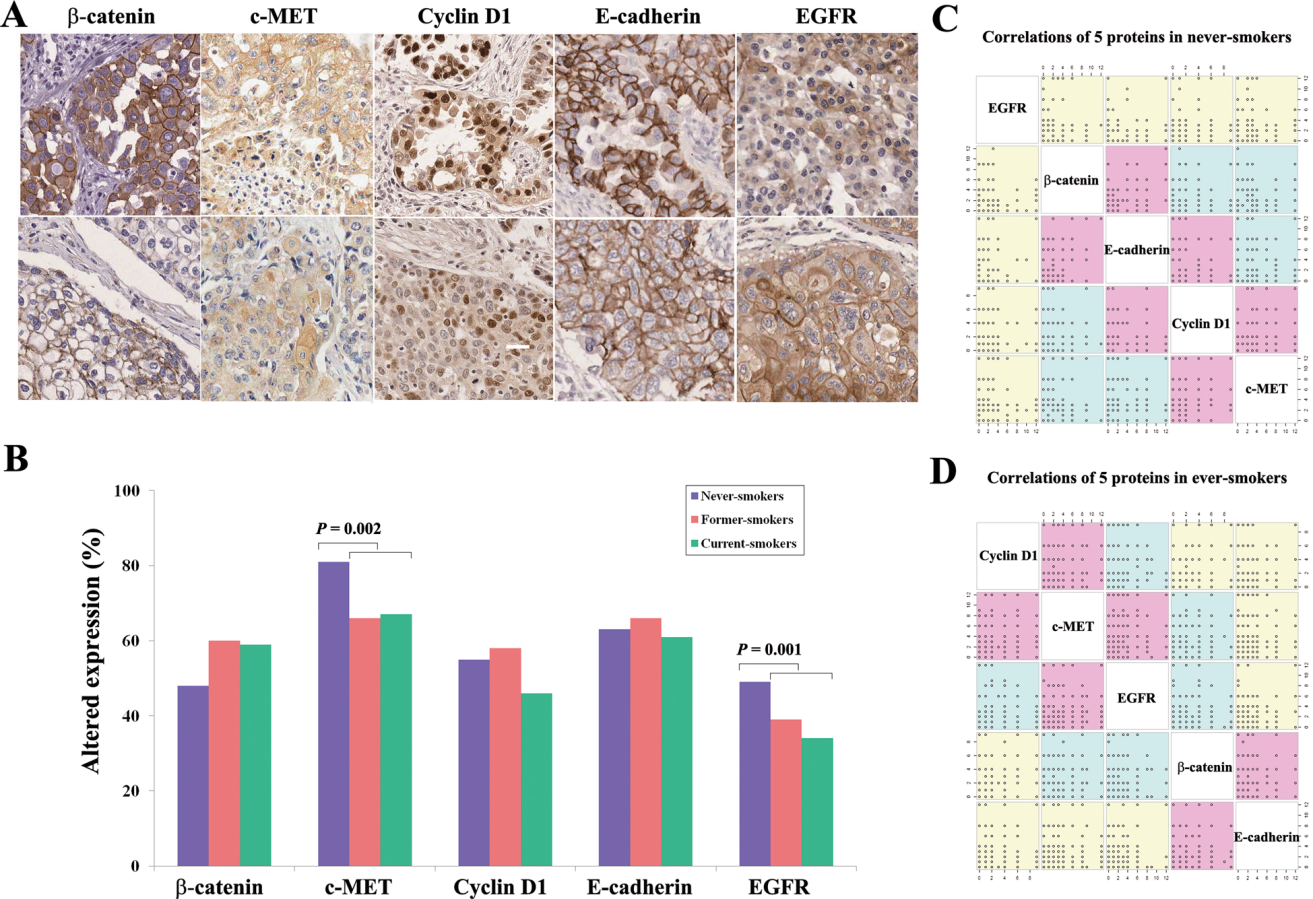
**Prevalence of altered expression of five proteins and their correlation according to smoking status. (A)** The expression levels of five proteins were analyzed using immunohistochemical staining. Representative examples of positive staining of the five proteins are shown in adenocarcinoma (upper) and squamous cell carcinoma (lower) (×200). **(B)** The prevalence of altered expression of individual proteins was compared according to smoking status. *P* values were based on Kruskal-Wallis test. **(C, D)** Correlation among the five proteins was analyzed using Spearman’s correlation coefficient in **(C)** never-smokers and **(D)** ever-smokers. Magenta color indicates *P* < 0.05.

The clinicopathological features of the 578 participants are summarized in Table 1. The expression of E-cadherin was found to be negative in 62% of them. Of those, the overexpression of β-catenin, c-MET, cyclin D1, and EGFR occurred in 55%, 72%, 51%, and 41% of patients, respectively. The positive immunohistochemical staining for the five proteins is shown in Figure 1A. Before analyzing if the effect of *RARβ2* hypermethylation on RFS in NSCLC was different according to smoking status and the possible interacting proteins, we first compared the prevalence of abnormal expression of five proteins among never-smokers, former-smokers, and current-smokers (Figure 1B). The prevalence of abnormal expression of β-catenin, cyclin D1, and E-cadherin was not significantly different according to smoking status, but the abnormal expression of c-MET (*P* = 0.002) and EGFR (*P* = 0.001) was found at a high prevalence in never-smokers compared to former- and current-smokers. For the former- and current-smokers, the prevalence of abnormal expression of the five proteins was similar, and therefore, we divided the patients into never-smokers and ever-smokers and analyzed the correlation of the expression levels of the five proteins in never-smokers (Figure 1C) and ever-smokers (Figure 1D). Expression levels of β-catenin were significantly correlated with those of E-cadherin in never-smokers (*P* = 0.02) and in ever-smokers (*P* = 0.01). In contrast, expression levels of cyclin D1 were significantly associated with those of EGFR in ever-smokers (*P* = 0.004) but not in never-smokers (*P* = 0.19). Based on these observations, it is likely that molecular alterations of some proteins and protein-protein interactions may differ between never-smokers and ever-smokers in NSCLC.

**Table 1:** **Clinicopathological features (** ***N*** **= 578)**

**Variable**	**Number (%)**
Age (years)^a^	60 ± 11
Tumor size (cm)^a^	4.1 ± 2.4
Pack-years of smoking^a^	32 ± 23
Sex	
Male	456 (79%)
Female	122 (21%)
Histology	
Adenoca	249 (43%)
Squamous	277 (48%)
Others	52 (9%)
Smoking status	
Never	151 (26%)
Former	145 (25%)
Current	282 (49%)
Pathologic stage	
I	258 (45%)
II	192 (33%)
III	122 (21%)
IV	6 (1%)
Differentiation^b^	
Well	96 (19%)
Moderate	301 (58%)
Poorly	113 (22%)
Undifferentiated	9 (2%)
Adjuvant chemotherapy	
No	509 (88%)
Yes	69 (12%)
Adjuvant radiotherapy	
No	451 (78%)
Yes	127 (22%)
Neo-adjuvant chemotherapy	
No	544 (94%)
Yes	34 (6%)
Neo-adjuvant radiotherapy	
No	555 (96%)
Yes	23 (4%)

Adenoca, adenocarcinoma; Squamous, squamous cell carcinoma.

^a^Values indicate mean ± standard deviation.

^b^Differentiation data are missing for 61 patients.

### The effect of *RARβ2* hypermethylation on RFS in never-smokers is different according to histology

**Figure 2:**
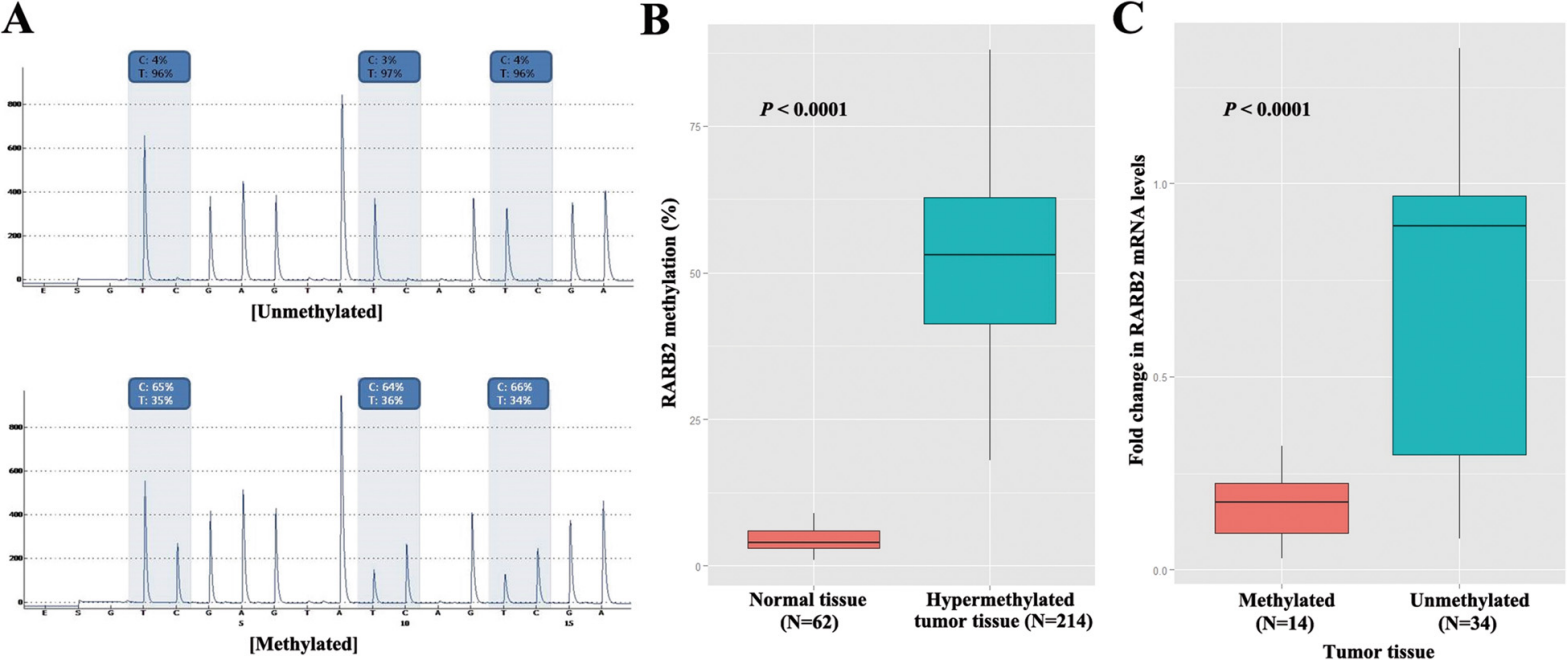
**Quantitative pyrosequencing analysis of** ***RARβ2*** **methylation. (A)** Methylation levels of *RARβ2* were quantitatively measured using pyrosequencing. The pyrograms of *RARβ2* show low levels of *RARβ2* in unmethylated CpGs (upper) and high levels in methylated CpGs (lower). **(B)** Methylation levels of *RARβ2* were compared between 62 normal control tissues from formalin-fixed paraffin-embedded tissue and 214 hypermethylated tumor tissues (*P* < 0.0001; Wilcoxon rank-sum test). **(C)** To determine if *RARβ2* hypermethylation is associated with transcriptional silencing, *RARβ2* mRNA levels normalized to GAPDH were compared between methylated (*N* = 14) and unmethylated (*N* = 34) fresh-frozen tissues. The fold change in *RARβ2* mRNA levels was significantly different between the two groups (*P* < 0.0001; Wilcoxon rank-sum test).

Methylation levels of *RARβ2* were quantitated in 578 formalin-fixed paraffin-embedded tissues using bisulfite pyrosequencing (Figure 2A). To define *RARβ2* hypermethylation, we analyzed the methylation levels of *RARβ2* using pyrosequencing in 62 normal formalin-fixed paraffin-embedded lung tissues. *RARβ2* hypermethylation was defined as methylation levels greater than or equal to 10%, because the methylation levels of *RARβ2* in normal tissues ranged between 2% and 9%. Based on the criteria, the methylation levels of *RARβ2* showed a significant difference between normal tissues and hypermethylated tumor tissues (*P* < 0.0001; Figure 2B; Wilcoxon rank-sum test). *RARβ2* expression was measured using quantitative reverse transcription PCR in fresh-frozen tumors and matched normal tissues from 48 NSCLC patients due to insufficient number of tissue samples. The fold change in *RARβ2* mRNA levels normalized to GAPDH was compared between tumor tissues with and without *RARβ2* hypermethylation (Figure 2C). Fourteen (29%) of 48 tumor tissues showed a higher methylation of *RARβ2* and very low mRNA levels. The fold change in *RARβ2* mRNA levels was significantly different between the groups (*P* < 0.0001; Wilcoxon rank-sum test).

**Figure 3:**
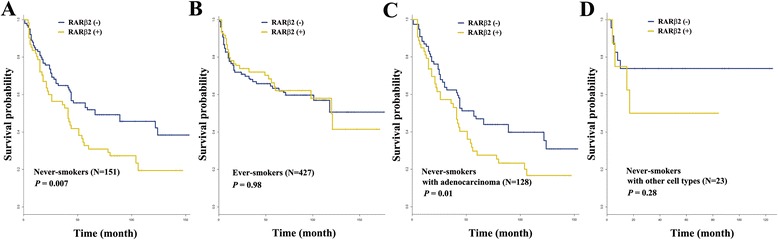
**Kaplan-Meier survival curves.** The effect of *RARβ2* hypermethylation on RFS was analyzed using a log-rank test in **(A)** 151 never-smokers, **(B)** 427 ever-smokers, **(C)** 128 never-smokers with adenocarcinoma, and **(D)** 23 never-smokers with other cell types. The minus and plus signs indicate the absence and presence of *RARβ2* hypermethylation, respectively. *P* values were based on the log-rank test.

Two hundred and sixty-eight patients (46%) had developed a recurrence during a median follow-up of 57 months; local recurrence in 18%, distant recurrence in 69%, and distant and local recurrence in 13%. To analyze the effect of *RARβ2* hypermethylation on RFS, data were stratified into two groups, never-smokers and ever-smokers. For 151 never-smokers, five-year RFS rates were significantly different according to methylation status of *RARβ2* (*P* = 0.007; Figure 3A): 31% for patients with *RARβ2* hypermethylation and 52% for those without *RARβ2* hypermethylation. For the 427 ever-smokers, *RARβ2* hypermethylation was not associated with RFS (*P* = 0.98; Figure 3B).

Lung cancer histology is known to be associated with smoking status: adenocarcinoma develops more frequently in never-smokers, whereas other cell types are more common in ever-smokers. This suggests that the relationship between RFS and *RARβ2* hypermethylation in never-smokers may be confounded by a histologic subtype. Therefore, the data was stratified by histologic subtypes, and RFS in never-smokers were further analyzed under this classification. For adenocarcinoma cases, five-year RFS rates were 27% and 46% in patients with *RARβ2* hypermethylation and in those without, respectively, and this difference was statistically significant (*P* = 0.01; Figure 3C). However, RFS in cases with other histologic subtypes was not found to be significantly different between patients with and without *RARβ2* hypermethylation (*P* = 0.28; Figure 3D).

### No heterogeneity of association between *RARβ2* hypermethylation and recurrence across the proteins

To adjust for effect modifiers or confounding factors in the relationship between *RARβ2* hypermethylation and RFS in never-smokers with adenocarcinoma, we evaluated if the population odds ratios between *RARβ2* hypermethylation and the risk of recurrence were uniform across the altered expression of individual proteins in never-smokers with adenocarcinoma. The Breslow-Day test for homogeneity of the odds ratio showed no evidence of heterogeneity in cases of *RARβ2* hypermethylation across the altered expression of β-catenin (*P* = 0.18), c-MET (*P* = 0.48), cyclin D1 (*P* = 0.27), E-cadherin (*P* = 0.95), and EGFR (*P* = 0.68). The altered expression of individual proteins might not be an effect modifier in the relationship between *RARβ2* hypermethylation and recurrence in our population. Therefore, we did not construct various contingency tables as if they had been drawn from distinct populations and computed a single summary measure rather than individual hazard ratio across the proteins for the 128 cases.

### No association between *RARβ2* hypermethylation and protein expression levels

**Figure 4:**
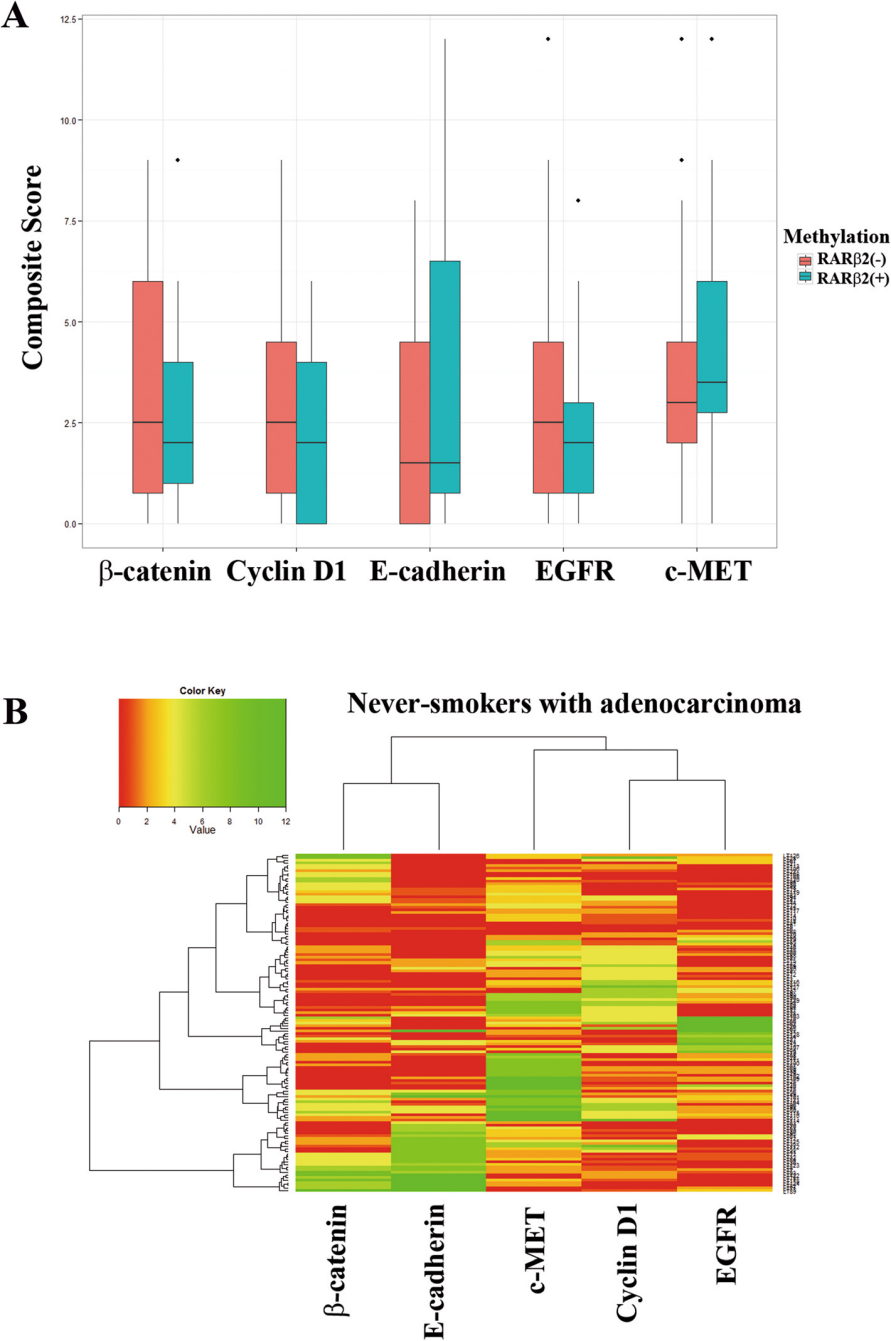
**Unsupervised clustering of protein expression in 128 never-smokers with adenocarcinoma. (A)** Boxplots of composite scores of each of the five proteins. Composite scores of the expression levels of the five proteins were compared according to methylation status of *RARβ2*. Salmon and dark turquoise bars indicate groups with and without *RARβ2* hypermethylation, respectively. Outliers are represented by black dots. **(B)** The hierarchical clustering of the expression patterns of the five proteins is shown. The scaled composite score of individual proteins is plotted in a red-green scale, with red indicating low expression and green indicating high expression. Each row represents individual tissues and each column represents the five proteins. The color in each cell reflects the expression levels of individual proteins in the corresponding tissue.

The five proteins analyzed in this study were considered as potential confounding factors in the relationship of *RARβ2* hypermethylation with RFS, since cyclin D1, β-catenin, and E-cadherin are known to interact with retinoid signaling. To evaluate if altered expression of individual proteins can be confounding variables in never-smokers with adenocarcinoma, we analyzed its association with *RARβ2* hypermethylation. Composite scores of individual proteins in never-smokers with adenocarcinoma were compared according to methylation status of *RARβ2* (Figure 4A). However, *RARβ2* hypermethylation was not associated with the composite scores of β-catenin (*P* = 0.65; Wilcoxon rank-sum test), c-MET (*P* = 0.30), cyclin D1 (*P* = 0.38), E-cadherin (*P* = 0.21), or EGFR (*P* = 0.07), suggesting that there may be no interaction between *RARβ2* and the five proteins in never-smokers with adenocarcinoma. Next, the univariate Cox proportional hazard model was applied to assess the independent effects of the five proteins on RFS in 128 never-smokers with adenocarcinoma. Tumor size, sex, histology, pathologic stage, and *RARβ2* hypermethylation showed a statistically significant association with RFS, but none of the individual proteins showed a significant association with RFS (Table 2).

**Table 2:** **Univariate Cox proportional hazards analysis of RFS in never-smokers with adenocarcinoma (** ***N*** **= 128)**

**Variable**	**HR (95% CI)**	***P*** **value**
Age (years)	0.98 (0.95 to 1.04)	0.58
Size (cm)	1.25 (1.12 to 1.40)	<0.0001
Sex	0.64 (0.40 to 0.97)	0.05
Differentiation	1.32 (0.97 to 1.75)	0.06
Pathologic stage	1.72 (1.41 to 2.08)	<0.0001
RARβ2	1.71 (1.16 to 2.57)	0.008
EGFR	0.82 (0.54 to 1.24)	0.35
c-MET	0.77 (0.46 to 1.25)	0.28
β-catenin	1.09 (0.72 to 1.65)	0.72
E-cadherin	1.14 (0.73 to 1.78)	0.54
Cyclin D1	1.10 (0.74 to 1.63)	0.63

HR, hazard ratio; CI, confidence interval.

### Multivariate Cox proportional hazards analysis of RFS

Finally, multivariate Cox proportional hazards analysis for 128 never-smokers with adenocarcinoma was performed to determine whether *RARβ2* hypermethylation was an independent prognostic factor for RFS, after controlling for the potential confounding effects of variables. The expression levels of the five proteins were not statistically associated with RFS in the 128 never-smokers with adenocarcinoma. However, given that retinoid signaling interacts with the proteins biologically, the proteins were included in the multivariate analysis. For more parsimonious model building, a data mining for the five proteins was conducted using an unsupervised hierarchical clustering and a heatmap showed the expression patterns of the five proteins (Figure 4B). The β-catenin was close to E-cadherin, and cyclin D1 was close to EGFR. The data were partitioned into three clusters (β-catenin/E-cadherin, c-MET, and cyclin D1/EGFR) and included in the multivariate analysis. A Cox proportional hazard model showed that RFS in 128 never-smokers with adenocarcinoma was found to be 2.19 times (95% confidence interval [CI] = 1.28 to 3.47; *P* = 0.009; Table 3) poorer than that in those without *RARβ2* hypermethylation. However, no protein clusters were associated with RFS.

**Table 3:** **Multivariate Cox proportional hazards analysis** ^**a**^ **of RFS according to** ***RARβ2*** **hypermethylation, stratified by histology, in never-smokers**

	***RARβ2*** **hypermethylation**	**HR**	**95% CI**	***P*** **value**
A. Adenocarcinoma	No	1.00		
(*N* = 128)	Yes	2.19	1.28 to 3.47	0.009
B. Other cell types	No	1.00		
(*N* = 23)	Yes	3.27	0.71 to 16.23	0.16

HR, hazard ratio; CI, confidence interval.

^a^Adjusted for age, sex, differentiation, tumor size, pathologic stage, histology, adjuvant chemotherapy, and protein clusters [(β-catenin/E-cadherin), c-MET, and (cyclin D1/EGFR)].

## Discussion

Several studies have suggested that retinoids may have harmful effects in the prevention of lung cancer. However, most studies about the tertiary chemopreventive effect of retinoids in surgically treated lung cancer have focused primarily on smokers, and relatively few studies have examined the chemopreventive effect of retinoids in never-smokers. Although tobacco smoking accounts for the majority of lung cancer as a presumed etiology, it is estimated that 15% of male and 53% of female lung cancer patients are lifelong never-smokers worldwide [21]. More and more studies have suggested that lung cancers occurring in never-smokers may be a distinctive disease due to unique molecular profiles [22-25]. To understand the effect of *RARβ2* hypermethylation on recurrence according to smoking status in NSCLC, we analyzed *RARβ2* hypermethylation and expression levels of five proteins in 584 NSCLCs. The proteins were adjusted as confounding factors in the relationship of *RARβ2* hypermethylation with recurrence.

Among the five proteins analyzed in this study, β-catenin and E-cadherin are known to interact with RAR [10-16]. β-catenin is a protein associated with the cytoplasmic tail of E-cadherin, and the level of β-catenin in the cytoplasm determines the activation of Wnt responsive genes. Nuclear β-catenin interacts with the TCF/LEF (T cell factor/lymphocyte enhancer factor) family of transcription factors and induces the transcription of genes involved in cell proliferation and/or inhibition of apoptosis. Thus, constitutive activation of β-catenin may be a critical step in tumorigenesis among divergent types of cancers. Retinoid signaling has been found to be a potent inhibitor for Wnt/β-catenin signaling in colon and breast cancer cells [13], neuronal cells [14], bronchial epithelial cells [15], and ES cells [16]. E-cadherin is also known to interact with RARs. In breast cancer cells, RA up-regulates the function of the invasion-suppressor complex E-cadherin/catenin [10]. Cadherin expression and function are necessary to mediate the effects of RA on adhesion and differentiation [13].

The interaction between retinoid signaling and cyclin D1 has also been reported in a variety of cancer cells. Retinoic acid triggers G_1_ cell cycle arrest through the degradation of cyclin D1 via the ubiquitin-proteasome pathway [17]. Ectopic expression of cyclin D1 sensitizes ER-positive breast cancer cells to a retinoic acid-induced mitochondrial death pathway [18]. The activity of the cyclin D1 reporter was reduced in response to retinoic acid in breast cancer cells independently of β-catenin/TCF signaling [13]. However, in this study, the altered expression of cyclin D1 was not associated with *RARβ2* hypermethylation (Figure 3A) or recurrence. These observations suggest that interaction between *RARβ2* signaling and E-cadherin/β-catenin or cyclin D1 may be different in never-smokers. In addition, *RARβ2* hypermethylation may affect RFS irrespective of an alteration of interacting proteins in never-smokers.

In this study, *RARβ2* hypermethylation was not associated with expression levels of the five proteins (Figure 4A). We further analyzed the association between *RARβ2* expression in 48 fresh-frozen tumor tissues and the expression of the other five proteins (data not shown). Twenty-six (54%) of 48 tumor tissues were found to lose *RARβ2* expression. No association was found between *RARβ2* expression and the expression of five proteins in the 48 samples. However, the association showed a different pattern in samples stratified by histology: *RARβ2* expression showed a reciprocal relationship with *EGFR* expression in adenocarcinoma and with c-MET expression in squamous cell carcinoma, suggesting that RARβ2 may interact with EGFR or c-MET in a tissue-dependent manner. Further study in a larger sample is required to clearly understand the relationship between RARβ2 and EGFR (or c-MET) according to histological subtypes.

Mutations in the epidermal growth factor receptor (EGFR) gene are more common in lung cancers from never-smokers compared to ever-smokers in both men and women. EGFR overexpression in this study was found in 71 (47%) of 151 never-smokers and was more frequent in adenocarcinoma (56%) than in squamous cell carcinoma (34%); results which were consistent with previous reports [19,20]. The c-MET is a proto-oncogene that codes for a protein known as hepatocyte growth factor receptor (HGFR), which has tyrosine kinase activity. In this study, overexpression of c-MET was found at a higher prevalence in never-smokers compared to ever-smokers (Figure 1B; *P* = 0.002). However, the altered expression of β-catenin, cyclin D1, and E-cadherin occurred at a similar frequency in never-smokers and ever-smokers. Based on these observations, some of the molecular alterations in never-smokers with NSCLC may be similar to those typically seen in lung cancer associated with tobacco smoking, but separate and distinct molecular features seem to exist in never-smokers.

*EGFR* overexpression in this study was not associated with RFS in never-smokers with adenocarcinoma (Table 2). To understand if *EGFR* mutations can affect RFS, we sequenced the exons 18, 19, and 21 of *EGFR* in 48 fresh-frozen tumor tissues (data not shown). The deletion in exon 19 or L858R missense mutation in exon 21 were found in 17 (34%) of 48 tumor tissues. The effect of the *EGFR* mutation on RFS was tested by Kaplan-Meier survival curve. Patients with an *EGFR* mutation showed a trend toward poor RFS, though it was not statistically significant (*P* = 0.08). The lack of an effect of the *EGFR* mutation on RFS in this study may have resulted from the small sample size or from the lack of targeted chemotherapy of tyrosine kinase inhibitors. The effect of the *EGFR* mutation on RFS in never-smokers with adenocarcinoma warrants further study in a large prospective cohort.

Is it reasonable to recommend the intake of retinoids for the chemoprevention of recurrence in never-smokers who have undergone surgical resection for NSCLC? The response to retinoic acid may be different according to a patient’s exposure to tobacco smoke. A significant restoration of *RARβ* expression and reduction of metaplasia were found in former-smokers treated with 9-cis-retinoic acid [9]. Treatment of 9-cis-RA in former-smokers modulated serum concentrations of insulin-like growth factor (IGF)-I, IGF binding protein (IGFBP)-3, and their molar ratio (IGF-I/IGFBP-3) [26]. In contrast to our previous finding that the hypermethylation of *RARβ2* gene in current-smokers had a beneficial effect on the development of SPLC in NSCLC (8), the present study suggests that *RARβ2* hypermethylation may be associated with poor RFS in never-smokers with adenocarcinoma. Taken together, the combination of retinoic acid and the demethylating agent is recommended for the prevention of recurrence irrespective of alteration of the five proteins in never-smokers with adenocarcinoma. In addition, retinoic acid may be beneficial for the primary prevention of lung cancer in never-smokers with high risk factors, such as particular genetic mutations predisposing them to cancer development and for the secondary prevention of disease progression in never-smokers with premalignant lesions.

This study was severely limited by several factors. First, the number of cases with other cell types rather than adenocarcinoma was very small. For the application of retinoic acid in a clinical practice, the effect of *RARβ2* hypermethylation on RFS in never-smokers with adenocarcinoma as well as other cell types should be further validated by prospective large-scale studies. Second, the molecular mechanisms underlying the finding that *RARβ2* hypermethylation contributes to poor RFS in never-smokers with adenocarcinoma should be studied. Third, other proteins interacting with RAR that were not analyzed in this study also need to be analyzed to clearly understand the effect of retinoic acid on RFS in never-smokers.

## Conclusions

*RARβ2* hypermethylation is known to occur in approximately 40% of NSCLCs. The present study suggests that *RARβ2* hypermethylation may be associated with poor RFS in never-smokers with adenocarcinoma.

## Methods

### Study population

A total of 578 formalin-fixed paraffin-embedded (FFPE) tissues were obtained from NSCLC patients undergoing surgical resection between May 1994 and September 2004 at the Samsung Medical Center in Seoul, Korea. The median duration of follow-up was 57 months. A written informed consent was obtained from the patients before surgery. This study was approved by the Samsung Medical Center (SMC) Institutional Review Board (IRB). Post-operative follow-up for the detection of local and distant recurrence was performed as previously described [2,8], and recurrence was evaluated from information obtained from our hospital’s electronic medical records and those from other hospitals, as of November 2013. All cases of cancer were classified based on the guidelines of the tumor-node-metastasis (TNM) staging system of the American Joint Committee on Cancer [27]. Never-smoker was defined as a person who has never smoked or who has smoked fewer than 100 cigarettes in his or her lifetime.

### Immunohistochemistry

Tissue microarrays (TMAs) of NSCLCs and immunohistochemical staining of the five proteins were conducted as previously described [28]. The sections were incubated overnight at 4°C with the following primary antibodies: β-catenin (clone 17C2; Novocastra, Buckinghamshire, UK) at 1:100 dilution, c-MET (3D4, Zymed, South San Francisco, CA, USA) at 1:200 dilution, cyclin D1 (SP4, Lab Vision, Fremont, CA, USA) at 1:50 dilution, E-cadherin (4A2C7, Zymed, South San Francisco, CA) at 1:100 dilution, or EGFR (1005, Santa Cruz, CA, USA) at 1:100 dilution. Detection of immunoreactivity by each antibody was performed by Envision^TM^+ peroxidase (Dako, Carpinteria, CA, USA). The peroxidase activity for each antibody was visualized by applying diaminobenzidine chromogen containing 0.05% hydrogen peroxide for 3 to 8 min at room temperature. Nuclei were then counterstained with Mayer’s hematoxylin, and negative controls were included in each run by omitting the primary antibody.

### Interpretation of immunohistochemical staining

The slides were read by two authors (EYC, JH), who were blinded to the clinical outcome of the patients, to reduce inter-observer variability. The correlation of scores between the two readers of immunohistochemical staining was calculated, and samples showing kappa values less than 0.20, poor inter-rater reliability, were removed from further analysis. Cyclin D1 was stained in the cytoplasm and nucleus, but only nuclear staining of cancer cells was considered positive. The immunoreactivity of EGFR and E-cadherin was found mainly in the membrane of neoplastic cells, and membrane staining was considered in scoring. The c-MET is localized in both the cell membrane and cytoplasm of cancer cells, and immunoreactivity in the cytoplasm and membrane was semi-quantitatively graded by considering the intensity of staining. Immunoreactivity for β-catenin was observed in the membrane, cytoplasm, and nucleus, and the intensity and proportion of positive staining cells only in the cytoplasm were evaluated for scoring.

The expression levels of five proteins were calculated by multiplying the intensity score (0, none; 1, weak; 2, moderate; 3, strong) and the proportion score of positive staining tumor cells (0, absent; 1, 0% to 10%; 2, 10% to 50%; 3, 50% to 80%; 4, >80%). Cutoff values of the composite scores for abnormal expression were determined by using an internal control consisting of 62 normal lung cores from 152 lung cancer blocks. To determine the cutoffs for abnormal expression of individual protein, we first analyzed the distribution of the composite scores for abnormal expression of β-catenin, cyclin D1, EGFR, E-cadherin, and c-MET proteins between the normal and tumor tissues. Based on the distribution, we set several cutoffs, compared false positives and false negatives at different cutoffs, and finally determined the cutoffs of the composite score that minimized false positives and false negatives. The β-catenin, c-MET, cyclin D1, and EGFR were considered to be overexpressed if the composite score was greater than or equal to 2. E-cadherin was defined as negative if the composite score of membrane staining was less than 2.

### Pyrosequencing

To microdissect tumor areas, we cut formalin-fixed, paraffin-embedded tissues into 10-μm-thick sections. Before DNA extraction, we performed hematoxylin-eosin staining to locate the tumor areas and microdissected areas containing at least 75% neoplastic tissue. Five hundred nanograms of genomic DNA was purified using the DNeasy Blood & Tissue Kit (Qiagen, Valencia, CA, USA) according to the manufacturer’s protocol and then bisulfite-converted using the EZ DNA methylation kit (Zymo Research, Irvine, CA, USA). The methylation levels of CpG islands at the P2 promoter region of the *RARβ2* gene were analyzed by pyrosequencing of bisulfite-treated DNA, as previously described by Lee et al. [29]. Pyrosequencing was carried out on a PSQ 96MD system with the PyroMark PCR kit (Cat. No. 978703, Qiagen), and data were analyzed using the Q-CpG software (v.1.0.9, Pyrosequencing AB, Uppsala, Sweden).

### Quantitative reverse transcription-PCR (qRT-PCR)

The expression of *RARβ2* was measured using TaqMan assays following the manufacturer’s instructions on the ABI 7500 Fast Real-Time PCR system (Applied Biosystems, Waltham, MA, USA). One microgram of total RNA was reverse transcribed using the SuperScript VILO cDNA synthesis kit (Cat. No. 11754-050, Invitrogen, Carlsbad, CA, USA), and the resulting cDNA was used for real-time PCR. The GAPDH was included as an internal reference for *RARβ2* expression. TaqMan primer sets (Hs00977141-mH for *RARβ2* and Hs0275899_g1 for *GAPDH*) were purchased from Life Technologies. TaqMan probe sequences for *RARβ2* and *GAPDH* were FAM-CCTGCCTGGACATCCTGATTCTTAG-MBG (110 bp) and FAM-GACTCATGACCACAGTCCATGCCAT-MBG (93 bp), respectively. *RARβ2* expression values were normalized to the reference by using the comparative Ct method.

### Statistical analysis

In the univariate analysis, the *t*-test (or Wilcoxon rank-sum test or Kruskal-Wallis test) was used for the analysis of continuous variables. Correlations among expression levels of the five proteins were compared using Spearman’s rank correlation coefficients. The prognostic significance of the five proteins and RARβ2 on recurrence-free survival (RFS) was assessed by Kaplan-Meier survival curves, and the significance of the difference in RFS between two groups was evaluated using the log-rank test. The hazard ratios of independent factors for RFS were estimated by multivariate Cox proportional hazard regression analysis. All statistical analyses were two sided with a 5% type I error rate. Expression patterns of the five proteins were recognized using an unsupervised hierarchical cluster analysis.

## Abbreviations

RFS: recurrence-free survival
NSCLC: non-small cell lung cancer
RA: retinoic acid
MSP: methylation-specific polymerase chain reaction
TMAs: tissue microarrays
EGFR: epidermal growth factor receptor
